# Cognitive Performance in a Subclinical Obsessive-Compulsive Sample 1: Cognitive Functions

**DOI:** 10.1155/2013/565191

**Published:** 2013-07-09

**Authors:** Thomas Johansen, Winand H. Dittrich

**Affiliations:** ^1^National Centre for Occupational Rehabilitation, Haddlandsvegen 20, 3864 Rauland, Norway; ^2^Research Center for Behavioral Economics, FOM Hochschule, Grüneburgweg 102, 60323 Frankfurt am Main, Germany

## Abstract

Individuals who are not clinically diagnosed with obsessive-compulsive disorder (OCD) but still display obsessive-compulsive (OC) tendencies may show cognitive impairments. The present study investigated whether there are subgroups within a healthy group showing characteristic cognitive and emotional performance levels similar to those found in OCD patients and whether they differ from OCD subgroups regarding performance levels. Of interest are those cases showing subclinical symptomatology. The results revealed no impairments in the subclinical OC participants on the neuropsychological tasks, while evidence suggests that there exist high and low scores on two standardised clinical instruments (Yale-Brown Obsessive Compulsive Scale and Cognitive Assessment Instrument of Obsessions and Compulsions) in a healthy sample. OC symptoms may diminish the quality of life and prolong sustainable return to work. It may be that occupational rehabilitation programmes are more effective in rectifying subclinical OC tendencies compared to the often complex symptoms of diagnosed OCD patients. The relationship between cognitive style and subclinical OC symptoms is discussed in terms of how materials and information might be processed. Although subclinical OC tendencies would not seem to constitute a diagnosis of OCD, the quality of treatment programmes such as cognitive behavioural therapy can be improved based on the current investigation.

## 1. Introduction

Obsessive thoughts and compulsive behaviours are not uncommon in the general healthy population. The prevalence and nature of obsessive-compulsive (OC) phenomena in nonclinical populations have revealed that up to 80% of the general population may experience intrusive, unpleasant, and unwanted thoughts similar to those seen in obsessive-compulsive disorder (OCD) [1]. These thoughts are commonly known as subclinical OC tendencies [2, 3]. However, these thoughts are generally not acted upon and not interpreted as harmful, unlike in individuals suffering from OCD, who give these thoughts meaning by attributing a cause to their recurrence [4] and interpret them as harmful and trying to resist them [1]. Abnormal and normal obsessions seem to share form and content similarities, but abnormal obsessions appear more frequent, more intense, and of longer duration and hence produce more discomfort [3, 5]. Similarly, there is also some evidence to suggest that continuities between abnormal and normal compulsions also exist, showing overlap in content, but eliciting more discomfort and being more intense and time consuming in OCD patients [2]. There are many nonclinical and completely normal manifestations of OC behaviours and thoughts, because perceptions of perfectionism, cleaning, and ethical and moral behaviour vary between people [6]. It appears that obsessions in OCD patients are misinterpreted as personally meaningful to an exaggerated extent, which constitutes a clinical diagnosis, while obsessions in subclinical OC populations are appraised in a way not implicating, for example, personal responsibility [7]. When directly comparing subclinical OC groups and nonclinical groups, the former seem to score higher in depression and anxiety [8, 9].

Subclinical OC tendencies have been proposed to be another dimension of OCD; that is, individuals who do not meet diagnostic criteria for OCD but still are vulnerable to neuropsychological deficits [10, 11]. Studying these OC tendencies will contribute to a better understanding of the nature of clinical OCD and the various forms in which it can be manifested [9] and it could have the potential to detect nonclinical individuals who are at risk of developing OCD, based on neuropsychological performance profiles [10]. In contrast to studying OCD patients, the effects of confounding factors such as medication and symptomatic state are avoided when assessing nonclinical individuals [12].

The first studies to systematically establish performance differences between subclinical and nonclinical individuals did not focus on cognitive deficits but rather relied mostly on behavioural experiments and questionnaire measures. Typically, healthy individuals (mostly students) were administered the Maudsley Obsessional Compulsive Inventory [13] and OC checkers were identified based on their endorsement of self-reported checking behaviour [14–16]. The results of these studies repeatedly confirmed impaired memory for actions in subclinical checkers compared to nonclinical checkers, a finding that has been replicated [17]. Memory impairments in the recall of past actions are thought to be related to checking compulsions, a lack of confidence in one's memory for actions, and the worry of forgetting (obsessions), which seem to drive the urge to carry out compulsive behaviours in subclinical individuals [15]. Similarly, the ability to distinguish between real and imagined events has also been found to be impaired in subclinical checkers [15], while the recall of verbal material has been reported to be intact [17]. A study comparing clinical OC checkers, subclinical checkers, and nonclinical checkers did not find differences in memory accuracy, but the two OC checker groups displayed reduced confidence levels in their memories [18]. A critique of the early memory research using subclinical populations was the lack of memory scenarios that induced a sense of personal responsibility [19], which may have produced different results or even stronger effects. 

Neuropsychological tasks have been used to investigate cognitive functioning in subclinical OC individuals. The performance on the Wisconsin Card Sorting Test (WCST) [20] revealed that subclinical checkers relative to nonclinical checkers made more total errors and perseverative errors and needed more time to complete the task [21]. This is in contrast to Mataix-Cols et al. [12], reporting no impairments in a group of subclinical individuals on the WCST as well as the Rey Auditory Verbal Learning Test [22], Trail Making Test part B [23], Controlled Oral Word Association Test [24], and the Stroop task [25]. On the other hand, they reported deficits on the Tower of Hanoi in subclinical individuals who needed more moves to solve the towers compared to healthy controls, an effect not related to clinical state, and it was suggested that a deficit in manipulating spatial information could be regarded as a trait marker in OCD [12]. The impairment on the Tower of Hanoi in subclinical individuals has later been confirmed, whereas the intact performance on the Rey Auditory Verbal Learning Test remained [11]. Furthermore, impairments have been demonstrated on a design fluency test in subclinical compared to nonclinical individuals even after controlling for mood, which suggest an inability to organise nonstructured material [26]. Memory abilities were investigated in subclinical and nonclinical checkers [27] and showed that subclinical checkers were impaired in response monitoring using the Self-Ordered Pointing Task [28] but displayed better performance compared to nonclinical checkers in reproducing abstract visuospatial designs after a 30-minute delay period. When controlling for trait anxiety symptoms, which was found to be significantly higher in the subclinical checkers, the effects of response monitoring were not as strong, but the visuospatial differences increased. 

 Other notable impairments in subclinical OC individuals have been found for manipulating information in visual working memory [29], lower scores on the Wechsler Memory Scale [14, 16, 30], set-shifting on the Object Alternation Test [31], the Delayed Alternation Test [32], and the TMT-B, but intact performance on the Controlled Oral Word Association Test and the WCST [33]. The Test of Everyday Attention [34] has revealed impairments in high-OC individuals [35], while subclinical individuals have been reported to perform to the same standard as nonclinical controls on the Stroop task [36]. Finally, an attentional bias for threatening information has been found in subclinical undergraduate students [37]. 

 Subclinical psychological symptoms, or similarly the notion of common mental disorders such as anxiety and depression, are major determinants of sick leave in the OECD countries [38]. Elucidating and better understanding the implications of subclinical psychological tendencies on the working life seem an avenue worth considering and are highlighted in the present study. 

 There seem to be two extreme cutoff points in subclinical OC research, one “liberal” and one “conservative”, and then everything in between [9]. This is based on the results from a large student population where the performance on the Padua Inventory [39] was used to group the participants into those scoring 1, 1.5, or 2 standard deviations above the mean. These three groups did not differ in most clinical symptomatology related to obsessions, anxiety, and depression, but they all scored higher compared to a matched nonclinical control group [9]. Also, ratings on the Yale-Brown Obsessive-Compulsive Scale (Y-BOCS) [40] in diagnosed OCD patients revealed that symptoms were still present after a two-year follow-up study [41], and similar findings have been proposed using the revised version of the Obsessive Compulsive Inventory in students [42]. 

The current study aimed to systematically investigate the cognitive profile in subclinical OC individuals on a range of cognitive tasks. Because of the limited research conducted on subclinical individuals and the lack of any established findings, it could be expected that the cognitive performance is only deficient to a minor degree in subclinical individuals, whereas in clinically diagnosed OCD individuals the cognitive processing may be detrimental to a major degree. Therefore, the performance of subclinical individuals may only be associated with a minor change in cognitive processing compared to OCD patients [43–48]. According to the scores on the Y-BOCS and the Cognitive Assessment Instrument of Obsessions and Compulsions (CAIOC) [43], a sample of healthy individuals was split into those scoring high (subclinical OC group) and low (nonclinical control group) in OC tendencies using relevant cutoff criteria. Although Mataix-Cols et al. [9] have suggested a range of reliable cutoff criteria to assess clinical morbidity in subclinical research, these may not be appropriate to apply in cognitive neuropsychological research, and it was decided to explore several criteria to assess how they impacted on neuropsychological performance.

## 2. Methods

### 2.1. Participants

There were 70 healthy individuals (47 female, 23 male) who participated, and the mean age was 37.2 years (standard deviation (SD) = 14.6). The participants who volunteered to take part were recruited from a student and a general population in the south east of England, UK, by newspaper, posted advertisements, and medical and ancillary staff from a local hospital in the south east of England, UK. Exclusion criteria constituted having experienced current or past history of DSM-IV-TR Axis I disorders according to assessment with the Mini International Neuropsychiatric Interview (MINI) [49]. The study was approved by the County Partnership NHS Trust Local Research Ethics Committee, UK. Data in this paper were obtained according to the Helsinki Declaration.

### 2.2. Materials

#### 2.2.1. Clinical and Psychological Background Measures

*MINI*. It is designed as a semistructured interview for the major Axis I psychiatric disorders in DSM-IV-TR and ICD-10. 

*Y-BOCS*. It is a commonly used clinical, semistructured interview which assesses the severity of obsessions and compulsions. The scale includes a 10-item symptom checklist designed to uncover the main target symptoms ranging from a total score of 0 to 40. The severity of these symptoms is evaluated in terms of the time (hours) spent on obsessions and compulsions, the distress and interference they cause, the degree of resistance to symptoms, and the control the patient has over them. A score below 16 indicates mild OCD, a score between 16 and 25 indicates moderate OCD, and a score higher than 25 indicates severe OCD.

*Montgomery-Åsberg Depression Rating Scale (MADRS) [50]*. It consists of ten statements, ranking from 0 to 6, where 6 is considered to be most associated with depression. This can be used to monitor a patient's state over time, and those scoring high for questions such as reduced appetite, pessimistic thought, and concentration difficulties would be most depressed. A score of 16 and above on the MADRS is the DSM-IV-TR criterion for depression. Maximum score on the MADRS is 60.

*State-Trait Anxiety Inventory (STAI) [51]*. It is a 40-item self-report questionnaire which includes separate measures of state and trait anxiety. The STAI clearly differentiates between the temporary condition of state anxiety and the more general and long-standing quality of trait anxiety. Typical state anxiety statements are as follows: I feel calm, I feel secure, and I am tense. Typical trait anxiety statements are worded similarly: I feel pleasant, I tire quickly, and I feel like crying. Maximum score on the STAI is 80.

*CAIOC*. It evaluates the cognitive and executive impairments that are hypothesised to underpin the impact of OCD symptoms on functioning, including obsessions and compulsions. The CAIOC was devised to fulfil the need for a brief, simple clinician, and patient-friendly instrument providing a reliable and valid dimensional measure of cognitive and clinical symptoms in patients suffering with OCD. It is designed to take less than 10 minutes to complete, with 18 questions ranked 0–6. In all cases 6 is considered to be most associated with functional impairment, and 0, the least on each assessment question. The CAIOC has been validated as a self-rated instrument but can also be administered by an experienced clinician. The final version of the CAIOC contains 13 items, reduced from 18 through the validation procedure. However, the 18-item version was used in the present study to capture all relevant functional impairments thought to be associated with OCD. The maximum score on the CAIOC-18 is 108. 

*Compulsive Personality Assessment Scale (CPAS) [52]*. It is based on the eight DSM-IV-TR factors for obsessive-compulsive personality disorder (preoccupation with details, perfectionism, workaholism, overconscientiousness, hoarding, need for control, miserliness, and rigidity). Symptoms which were present in adolescence or early adulthood as well as at the time of rating should be considered and quantified. Maximum score on the CPAS is 32.

*Sheehan Disability Scale (SDS) [53]*. It is a self-rated scale assessing perceived impairment along the three dimensions work, social life and leisure activities, and family life and home responsibilities. Maximum score on the SDS is 30.

*Locus of Control (LoC) [54]*. It is assessing the extent to which individuals believe that they can control events that affect them. Individuals with a high internal locus of control believe that events result primarily from their own behaviour and actions. Those with a high external locus of control believe that factors such as other people, chance, and fate primarily determine events. Those with a high internal locus of control have better control of their behaviour and tend to exhibit more political behaviours than externals. It is expected that OCD patients may endorse external statements more than internal because they are suggested to exhibit a dysfunctional belief system where factors not under their control may mediate the obsessions and compulsions. The maximum external score is 23.

*National Adult Reading Test (NART) [55]*. It is a word recognition test that utilises the high correlation between reading ability and intelligence in the general population. The test is composed of a list of 50 irregular words (pronunciation does not follow the normal phonetic rules) printed in order of increasing difficulty. The predicted verbal IQ score is determined from the number of reading errors the individual makes. The NART is a valid and reliable estimate of pre-morbid verbal intelligence.

#### 2.2.2. Neuropsychological Measures

The following neuropsychological tasks from the Cambridge Neuropsychological Test Automated Battery (CANTAB) [56] were administered: affective go/no-go (AGN; assesses information processing biases for positive and negative stimuli), Cambridge gambling task (CGT; assesses impulse control and risk taking in decision making), intra/extradimensional set shift (IED; tests rule acquisition and attentional set shifting), simple reaction time (SRT; measures speed of response to a single stimulus), choice reaction time (CRT; measures speed of choice responses), spatial recognition memory (SRM; tests recognition memory for spatial locations), spatial working memory (SWM; tests working memory and strategy use), and stockings of Cambridge (SOC; assesses spatial planning and motor control). Also, administered were the Iowa Gambling Task (IGT; assesses decision making under ambiguous conditions) [57] and the paper and pencil verbal and nonverbal reasoning tests [58]. All tests apart from the verbal and nonverbal reasoning tests were administered on a Hewlett-Packard portable laptop with a screen size of 330 millimetres in width and 210 millimetres in height. A Magic-Touch sensitive screen matching the size of the laptop screen and a press pad were used to record the participants' responses on the CANTAB tasks, whereas the laptop keyboard was used for the IGT.

### 2.3. Procedure

The participants were rated on clinical measures (Y-BOCS, MADRS, and CPAS) and the NART. The self-rated clinical and psychological measures (STAI, CAIOC, SDS, and LoC) were completed on the day of testing. For each participant the neuropsychological testing was completed in one session. If this was not possible, a new testing session was arranged within the same week or the week after. In total, the screening interview (MINI), clinical ratings, and self-rated measures took 45 minutes, and the neuropsychological testing lasted for 2 hours and 30 minutes including breaks. 

Two measures were used to allocate participants to either the subclinical group or the nonclinical group. To warrant inclusion in either the subclinical or nonclinical group, only participants scoring above or below the sample mean on both the Y-BOCS and the CAIOC were included. This decision was taken because group inclusion in the subclinical domain should be based on a wide range of OC tendencies.

### 2.4. Data Analysis

Scores on the background variables Y-BOCS, CAIOC, STAI-state, STAI-trait, and SDS were analysed using the nonparametric Mann-Whitney *U* test because of unequal variance in the subclinical and nonclinical groups. A logarithmic (base 10) transformation was performed for initial thinking times on the SOC to reduce skewness and improve normality. The study used a mixed design, with the between-subjects factor group (subclinical/nonclinical) and within-subject factors (different conditions of neuropsychological tasks, IGT: block (disadvantageous card selections in block 1/2/3/4/5); CGT: ratio of coloured boxes (6 : 4/7 : 3/8 : 2/9 : 1) and condition (ascending/descending); SWM: difficulty level (4/6/8 box-search problems); SOC: number of moves prior to obtaining the correct solution for each difficulty level (easy/hard), initial thinking time prior to moving the first ball for each difficulty level (easy/hard), and subsequent thinking time until obtaining the correct solution for each difficulty level (easy/hard); AGN: valence (happy/sad/neutral) and block (shift/nonshift)). When appropriate, the data from the neuropsychological task performance were submitted to repeated measures analysis of variance (ANOVA), one-way ANOVA, or independent samples *t*-tests. The significance level alpha was set at 0.05 and only *P* values below the alpha level are reported. The partial eta squared (*η* ^2^) was used as an effect size measure, which indicates the proportion of total variability attributable to a factor when analysing three or more groups. When comparing two groups, Cohen's [59] suggestion that effect sizes of .20 are small, .50 are medium, and .80 are large was followed. When the sphericity assumption was violated for repeated measures ANOVA, the Huynh-Feldt was reported. The categorical variables gender and handedness were subject to Pearson's chi-square analyses. Correlations between the Y-BOCS, MADRS, STAI-state, STAI-trait, CAIOC, and the neuropsychological task measures were examined using Pearson product-moment correlation coefficient.

## 3. Results

The results of the background measures are described first, followed by the neuropsychological performance in the subclinical and nonclinical groups.

### 3.1. Background Characteristics in the Subclinical and Nonclinical Group

The 70 healthy participants had a mean score of 2.3 (SD = 2.0) on the Y-BOCS and 27.9 (SD = 14.2) on the CAIOC. From the 70 participants, 26 (37% of the sample) met the criteria for inclusion in the subclinical group (i.e., scoring above the sample mean on both the Y-BOCS and the CAIOC) and 23 (33% of the sample) fulfilled the criteria for a grouping in the nonclinical control group (i.e., scoring below the sample mean on both the Y-BOCS and the CAIOC). Consequently, 21 (30% of the sample) participants were excluded from any further analysis. 

From Table 1, it can be seen that the subclinical group scored significantly higher compared to the nonclinical group in ratings on the Y-BOCS, *t*(47) = 11.585, *P* < .001, CPAS, *t*(47) = 3.984, *P* < .001, and CAIOC, *z* = −5.243, *P* < .001. A paired-samples *t*-test confirmed that trait anxiety in the subclinical group (M = 38.5, SD = 9.4) was significantly higher compared to state anxiety (M = 33.7, SD = 8.7), *t*(25) = 2.794, *P* = .010. 

### 3.2. Decision-Making

On the IGT, each participant made 100 card selections choosing from four different card decks. The number of times the disadvantageous cards were selected in each of five blocks was counted to assess whether the decision-making selection was random or deliberate. A two-way repeated measures ANOVA revealed that the number of disadvantageous card selections in each block was broadly similar in both groups (Table 2).

On the CGT, three separate three-way repeated measures ANOVAs were conducted to investigate the performance of the two groups for percentage of rational decisions, deliberation time, and percentage of points gambled in the ascending and descending conditions as a function of the ratio of coloured boxes. There were main effects of colour ratio for rational decisions, *F*(3,45) = 5.045, *P* = .004, *η*_*P*_^2^ = .252, deliberation time, *F*(3,45) = 7.813, *P* < .001, *η*_*P*_^2^ = .342, and points gambled, *F*(3,45) = 34.414, *P* < .001, *η*_*P*_^2^ = .696. For points gambled, there was also a main effect for condition, *F*(1,47) = 61.561, *P* < .001, *η*_*P*_^2^ = .567. Therefore, both groups displayed comparable decision-making behaviour, tending to be more rational, deliberate shorter and increase the bets at the more favourable ratios of red and blue boxes compared to the unfavourable (Table 2).

### 3.3. Visuospatial Working Memory

On the SRM the subclinical participants (M = 87.1%, SD = 7.5) recognised significantly more correct locations compared to the nonclinical participants (M = 81.1%, SD = 10.4), *t*(47) = 2.340, *P* = .024. On the SWM, a two-way repeated measures ANOVA was conducted to compare the performance in the two groups for between-search errors at the 4, 6, and 8 box difficulty levels. As expected, results revealed a main effect for difficulty level, *F*(1.45,68.06) = 84.122, *P* < .001, *η*_*P*_^2^ = .642 (sphericity assumption violated, Huynh-Feldt reported), indicating that more errors were made when the level of difficulty increased (Table 3).

### 3.4. Attention

On the SRT and CRT, the two groups did not differ in mean correct reaction time and percentage of correct trials (Table 4).

### 3.5. Planning and Behavioural Organisation

On the SOC, the performance in the two groups was examined with three separate two-way repeated measures ANOVAs for the easy and hard difficulty levels related to the number of moves needed prior to obtaining the correct solution and initial and subsequent thinking time. For number of moves, the result revealed a main effect for difficulty level, *F*(1,47) = 734.983, *P* = .001, *η*_*P*_^2^ = .940, indicating that participants needed more moves to solve the problems at the hard difficulty level compared to the easy. Main effects for difficulty level related to initial, *F*(1,47) = 60.817, *P* < .001, *η*_*P*_^2^ = .564, and subsequent thinking time, *F*(1,47) = 21.357, *P* < .001, *η*_*P*_^2^ = .312, were also identified and indicated that participants required, as expected, more initial and subsequent thinking time to solve the problems at the hard difficulty level compared to the easy (Table 5).

### 3.6. Affective Go/No-Go

On the AGN, three separate three-way repeated measures ANOVAs were conducted to compare the performance in the two groups for reaction time, number of false alarm responses, and target misses to happy, sad, and neutral words in the shift and nonshift block conditions (Table 6). For reaction time, the results revealed a main effect for valence, *F*(2,46) = 33.395, *P* < .001, *η*_*P*_^2^ = .592, indicating that participants had faster reaction times to happy and sad compared to neutral target words as well as responding faster to happy compared to sad target words. For false alarm responses, the results revealed a main effect for valence, *F*(2,46) = 21.338, *P* < .001, *η*_*P*_^2^ = .481, indicating that participants made more false alarm responses to neutral distractors compared to happy and sad as well as to happy distractors compared to sad. For misses, the results revealed a main effect for valence, *F*(2,46) = 15.425, *P* < .001, *η*_*P*_^2^ = .401, indicating that participants missed more neutral targets compared to happy and sad as well as missing more happy targets compared to sad.

## 4. Discussion

Remarkably, few systematic studies have so far reported the relationship between subclinical OC tendencies in healthy individuals and the performance on standardised neuropsychological tasks. It was found that, in a healthy population, there exist high and low scorers on clinical measures, which constituted a subclinical group and a nonclinical control group. Contrary to general expectations, the subclinical group performed to the same standard compared to the nonclinical control group on all cognitive tasks. In fact, the only performance difference that was revealed indicated a superior visuospatial recognition ability in the subclinical compared to the nonclinical participants. The significantly higher OC symptom severity observed in the subclinical compared to the nonclinical group extend previous results [8, 9, 42].

The present study is believed to be the most comprehensive investigation of neuropsychological performance in subclinical and nonclinical individuals to date employing a wide range of cognitive tasks. The findings may generalise more reliably to the healthy population showing OC tendencies because the age range in both the subclinical and the nonclinical group was from 18 to 77 years. This is in contrast to previous studies recruiting undergraduate students only [11, 12, 60]. Previously, the literature had presented intact [12] and impaired [21] findings on the WCST and evidence for deficits in subclinical individuals on the Tower of Hanoi [11, 12], the Object Alternation Test, and the Delayed Alternation Test [33]. Despite the null findings in the present study, correlation analyses were conducted and confirmed that neuropsychological performance in the subclinical individuals was not affected by depression and anxiety symptoms. Future studies might consider including highly anxious and depressed individuals as subclinical control groups to investigate whether differences in cognitive functioning relative to subclinical OC individuals exist to fully account for a wider range of clinical symptoms severity. 

The present results do not support the notion of endophenotypic markers to be present in OCD, that is, trait markers that can predict cognitive impairment and that are postulated to be linked to neural substrates that can reveal who are genetically at risk of developing mental disorders [61, 62]. Following this concept, it is claimed that some individuals, such as first degree relatives of patients with OCD [63] and OCD patients whose symptoms improved following successful pharmacological treatment [64], demonstrated impairments on selective neuropsychological tasks and could therefore be vulnerable because cognitive deficits may reflect trait markers rather than state markers [62]. The notion of an endophenotype is one that is heritable and is assumed to be present at a higher rate in nonaffected family members than in the general population [61]. Endophenotypes are claimed to be open to genetic dissection, and hence, more reliable components associated with psychiatric illnesses than the diagnostic categories themselves [61]. However, the present results support the proposal in a recent review that endophenotype markers are no more reliable measures of genetic susceptibility to develop psychiatric disorders than the clinical and behavioural symptoms themselves [65]. That is, if OC trait markers negatively affect neuropsychological performance, one would assume that the current subclinical group might have performed worse than the nonclinical control group. Moreover, the OC (Y-BOCS) and functional impairment tendencies (CAIOC) could affect cognitive processing, but not to a degree detrimental to cognitive functioning, which is in contrast to how cognitive processing can manifest itself to a much more damaging degree in OCD patients [43–48]. Subclinical groups within the healthy population seem to be distinguished from OCD patients based on their neuropsychological performance on selective cognitive tasks only on the basis of different degrees of information processing capabilities. Furthermore, the impairment established on the WCST in subclinical checkers in Goodwin and Sher [21] demonstrated that these individuals' performance was within the range of normal functioning, which supports the notion that cognitive processing is altered in subclinical individuals but not to a degree which seems damaging to cognitive abilities. Since obsessions and compulsions in diagnosed OCD patients are experienced as more intense and discomforting [3], the neuropsychological performance may in some instances depend on the clinical state in OCD patients showing cognitive impairment [66].

In occupational rehabilitation, it is well known that recovering patients may have subclinical tendencies in relation to various psychological disorders [67]. A range of factors have been identified to be important in prolonging the time to return to work in people who have been on long-term sickness absence. The most common are stress, fatigue, anxiety, depression, and musculoskeletal and chronic pain [67, 68], and consequently, these are the most common medical reasons for sick leave and disability pension [38, 69]. Here, we briefly mention the link between reduced work ability, return to work, and common mental disorders not dissimilar to subclinical psychological disorders as highlighted in the present study.

Subclinical OC tendencies can be viewed as a particular cognitive style mediated by particular ways of thinking and coping, affecting quality of life. OC tendencies can be characterised by stereotypical and narrow ways of processing information, which could lead to particular ways of carrying out behavioural actions, resembling compulsions. Findings from Burns and Fedewa [70] could be used to support this idea as they viewed perfectionism as a thinking style. Perfectionism can be characterised by ruminations over fears of making mistakes, which leads to failures being overexaggerated by the obsessional thoughts [71]. One can also distinguish between positive and negative perfectionism, justifying perfectionism as a thinking style [70]. OC tendencies could be perceived as a cognitive style in the same way as with perfectionism. There seem to be several definitions of thinking styles, varying slightly in their meaning, but Riding and Cheema [72] suggested two distinct dimensions of thinking styles, defining one as the “wholist-analytic style dimension” in which an individual tends to organise information in wholes or parts [73, page 316], and the other as “verbal-imagery style dimension” in which an individual is inclined to represent information during thinking verbally or in mental pictures [73, page 316]. These definitions could be incorporated to explain OC tendencies as it could be assumed that the verbal-imagery style dimension seems to fit with the characteristics of obsessive thinkers. Moreover, the cognitive thinking style framework outlined here is worth following up considering that in a large epidemiological survey of 2200 respondents, it was revealed that approximately 22–26% suffered from obsessions and compulsions, but only 0.6% received a formal DSM-IV diagnosis of OCD after clinical reappraisal [74]. This indicates that subclinical tendencies seem widespread in the general population. Therefore, exploring the relationship of obsessive thinking styles and holistic versus verbal styles in greater detail is worthwhile.

## 5. Conclusions

The cutoff scores to constitute an inclusion in the subclinical and nonclinical groups were conservative estimations, because only those individuals who scored either above or below the mean on both the Y-BOCS and the CAIOC were included. This was done to ensure that the individuals forming the subclinical group reflected true OC and functional impairment tendencies. It has also been demonstrated that a range of clinical inclusion criteria can be used in subclinical research [9]. Therefore, an even stricter cutoff point was also applied where inclusion in the two groups was determined by those individuals scoring above the 75th (subclinical) and below the 25th (nonclinical) percentile of the distribution on both the Y-BOCS and the CAIOC. However, following these cutoff criteria none of the results changed, which may be due to a small sample size and consequently a lack of power. For prospective studies, it is recommended that the question of OC tendencies in a subclinical sample is investigated with a larger sample size applying a more extreme cutoff criterion comparing the two groups, for example, using the upper and lower quartile of the distribution. This may lead to more reliable criteria elucidating cognitive performance differences between subclinical nonclinical individuals.

Overall, OC and functional impairment tendencies in the subclinical group were not detrimental to cognitive performance. The current study has provided new knowledge about cognitive functioning in subclinical individuals. These preliminary results can be used in prospective studies to form both new and specific predictions about subclinical behaviour related to cognitive functioning and furthermore, to tailor more effective cognitive behavioural therapy programmes for subclinical OC individuals.

## Figures and Tables

**Table 1:** Demographic, clinical, and psychological background characteristics in the sub-clinical and non-clinical groups.

Variable	Sub-clinical (*n* = 26)		Non-clinical (*n* = 23)		*P*
	M	SD	M	SD	
Age (years)	35.5	13.0	41.3	15.1	
Education (years)	3.8	2.0	4.5	2.0	
NART	115.4	5.3	116.6	7.0	
Y-BOCS^#^	4.2	1.3	0.6	0.8	<.001
MADRS	3.9	2.4	3.5	3.1	
STAI-state^#^	33.7	8.7	31.7	11.7	
STAI-trait^#^	38.5	9.4	33.5	9.0	
CAIOC^#^	38.1	12.9	17.9	7.1	<.001
CPAS	7.5	3.3	4.0	2.8	<.001
SDS^#^	4.5	5.1	1.6	2.5	
LoC	12.0	3.6	11.6	2.8	

Note. CAIOC: cognitive assessment instrument of obsessions and compulsions; CPAS: compulsive personality assessment scale; LoC: locus of control; MADRS: montgomery-Åsberg depression rating scale; SDS: sheehan disability scale; STAI: state-trait anxiety inventory; Y-BOCS: Yale-Brown obsessive compulsive scale; ^#^nonparametric Mann-Whitney *U* test used because of unequal variance in the two groups.

**Table 2:** Means and standard deviations in the sub-clinical and non-clinical groups for the IGT and CGT task measures.

Variable	Sub-clinical (*n* = 26)		Non-clinical (*n* = 23)		*P*
	M	SD	M	SD	
IGT^#^					
Block 1	10.1	4.7	10.4	3.2	
Block 2	8.6	4.8	10.0	5.0	
Block 3	8.1	4.4	9.7	4.7	
Block 4	9.1	4.4	10.0	4.1	
Block 5	9.0	4.5	9.9	4.7	
CGT^#^					
Rational 6 : 4 (%)	94.5	8.5	92.1	11.3	
Rational 7 : 3 (%)	94.5	11.9	94.6	6.9	
Rational 8 : 2 (%)	96.9	6.4	95.9	7.4	
Rational 9 : 1 (%)	98.1	3.4	98.4	3.9	
Deliberation 6 : 4 (ms)	2246	867	2427	948	
Deliberation 7 : 3 (ms)	2028	676	2271	731	
Deliberation 8 : 2 (ms)	1930	590	1932	468	
Deliberation 9 : 1 (ms)	1878	766	1946	581	
Points gambled 6 : 4 (%)	47.7	13.7	44.9	14.9	
Points gambled 7 : 3 (%)	58.3	14.4	54.8	16.5	
Points gambled 8 : 1 (%)	68.0	15.4	65.9	15.5	
Points gambled 9 : 1 (%)	72.3	17.0	68.1	16.6	

Note. ms: milliseconds; ^#^there was no difference in decision-making performance between the two groups (IGT = CGT).

**Table 3:** Means and standard deviations in the sub-clinical and non-clinical groups for the SRM and SWM task measures.

Variable	Sub-clinical (*n* = 26)		Non-clinical (*n* = 23)		*P*
	M	SD	M	SD	
SRM					
Recognition (%)	87.1	7.5	81.1	10.4	.024
Latency (ms)	2265	640	2361	800	
SWM					
Errors 4 boxes	0.7	1.3	1.0	2.3	
Errors 6 boxes	6.5	6.6	6.3	7.0	
Errors 8 boxes	17.0	11.5	19.3	13.9	
Strategy	31.9	6.4	33.3	4.8	
Latency (ms)	757	259	782	284	

Note. ms: milliseconds.

**Table 4:** Means and standard deviations in the sub-clinical and non-clinical groups for the SRT and CRT task measures.

Variable	Sub-clinical (*n* = 26)		Non-clinical (*n* = 23)		*P*
	M	SD	M	SD	
SRT					
Reaction time (ms)	258	55	257	43	
% correct	97.8	1.9	98.4	1.4	
CRT					
Reaction time (ms)	309	45	325	56	
% correct	98.7	1.2	98.8	1.2	

Note. ms: milliseconds.

**Table 5:** Means and standard deviations in the sub-clinical and non-clinical groups for the IED, SOC, verbal, and nonverbal reasoning task measures.

Variable	Sub-clinical (*n* = 26)		Non-clinical (*n* = 23)		*P*
	M	SD	M	SD	
IED					
Extradimensional shift stage	12.9	5.8	16.1	8.3	
Intradimensional shift stage	7.3	0.9	7.6	1.8	
Final stage	8.9	0.2	9.0	0.0	
Latency (ms)	1361	399	1394	308	
SOC					
Moves easy	2.6	0.2	2.6	0.2	
Moves hard	5.9	0.9	5.9	0.9	
Initial thinking time easy (ms)	2321	1193	3291	1998	
Initial thinking time hard (ms)	7017	5702	8320	5272	
Subsequent thinking time easy (ms)	545	777	421	873	
Subsequent thinking time hard (ms)	1071	1284	1319	1319	
Perfect solutions	8.7	2.1	8.9	2.0	
Reasoning					
Verbal	35.3	3.1	36.1	2.4	
Nonverbal	13.6	2.3	14.3	2.8	

Note. ms: milliseconds.

**Table 6:** Means and standard deviations in the sub-clinical and non-clinical groups for the AGN task measures.

Variable	Sub-clinical (*n* = 26)		Non-clinical (*n* = 23)		*P*
	M	SD	M	SD	
Happy targets (ms)	487	80	464	53	
Sad targets (ms)	489	68	478	52	
Neutral targets (ms)	537	98	517	72	
Happy false alarms	12.5	6.3	13.9	7.0	
Sad false alarms	10.3	6.6	11.6	6.7	
Neutral false alarms	16.4	7.3	17.7	6.5	
Happy misses	8.9	7.4	6.9	5.3	
Sad misses	6.2	6.9	6.0	4.7	
Neutral misses	12.7	9.9	11.2	8.0	

Note. ms: milliseconds.
